# CUD003, a Novel Curcumin Derivative, Ameliorates LPS-Induced Impairment of Endothelium-Dependent Relaxation and Vascular Inflammation in Mice

**DOI:** 10.3390/ijms26188850

**Published:** 2025-09-11

**Authors:** Hirokazu Matsuzaki, Anna Arai, Meiyan Xuan, Bo Yuan, Jun Takayama, Takeshi Sakamoto, Mari Okazaki

**Affiliations:** 1 Laboratory of Pharmacology, Faculty of Pharmaceutical Sciences, Josai University, Saitama 350-0295, Japan; ma-tsu@josai.ac.jp (H.M.); gyd2302@josai.ac.jp (A.A.); yuanbo@josai.ac.jp (B.Y.); 2 Laboratory of Organic and Medicinal Chemistry, Faculty of Pharmaceutical Sciences, Josai University, Saitama 350-0295, Japan; genbien@josai.ac.jp (M.X.); takayama@josai.ac.jp (J.T.); sakamoto@josai.ac.jp (T.S.)

**Keywords:** curcumin derivative, endothelial dysfunction, lipopolysaccharide, inflammation, oxidative stress, endothelial nitric oxide synthase

## Abstract

Endothelial dysfunction is closely linked to inflammation and oxidative stress and ultimately contributes to the development of cardiovascular diseases. Lipopolysaccharide (LPS), a major component of Gram-negative bacteria, induces vascular inflammation and oxidative damage in experimental models. Curcumin (Cur), a polyphenol from *Curcuma longa*, is well known for its anti-inflammatory and antioxidant properties. In this study, we examined the protective effects of CUD003, a novel synthetic Cur derivative, on the LPS-induced impairment of endothelium-dependent relaxation in the thoracic aorta of mice. Male ICR mice were pretreated with CUD003 or Cur (3 or 10 mg/kg, p.o.) 30 min prior to LPS injection (10 mg/kg, i.p.). Twenty-four hours after LPS injection, vascular reactivity was assessed in isolated aortic rings by evaluating vasorelaxation and vasoconstriction responses. LPS markedly impaired acetylcholine-induced vasorelaxation in the phenylephrine (PE)-precontracted aortic rings, while PE-induced contraction and sodium nitroprusside-induced relaxation were preserved, indicating that LPS impaired endothelium-dependent relaxation without affecting smooth muscle function. Immunohistochemical analysis revealed a reduction in eNOS expression and elevated levels of TNF-α, COX-2, O_2_^−^, and malondialdehyde, indicating enhanced inflammation and oxidative stress in the aorta. Pretreatment with CUD003 (10 mg/kg) significantly ameliorated these changes and showed superior protective effects compared to the same dose of Cur. These findings suggest that CUD003 protects against LPS-induced vascular dysfunction and suppresses inflammation and oxidative stress, supporting its potential as a preventive candidate against vascular inflammation and dysfunction.

## 1. Introduction

Endothelium-dependent vasodilation is a crucial physiological process that maintains vascular homeostasis and regulates blood flow. This response is primarily mediated by endothelial nitric oxide synthase (eNOS)-derived NO and constitutes a fundamental mechanism for the regulation of vascular tone, the control of blood flow, and the protection of vascular integrity. The impairment of this function, commonly characterized by endothelial dysfunction, is associated with various cardiovascular diseases such as atherosclerosis, hypertension, and sepsis-induced vascular damage [1,2,3,4]. Emerging evidence indicates that vascular inflammation is a central contributor to endothelial dysfunction and cardiovascular pathogenesis. The activation of endothelial cells and immune cells by inflammatory stimuli leads to excessive production of reactive oxygen species (ROS) and proinflammatory cytokines, such as tumor necrosis factor-alpha (TNF-α) and interleukin-6 (IL-6) [5,6]. These inflammatory mediators promote oxidative stress primarily through nicotinamide adenine dinucleotide phosphate (NADPH) oxidase activation, impair eNOS function, and reduce NO bioavailability, collectively disrupting endothelial function and vascular tone regulation [6,7].

Lipopolysaccharide (LPS), a major component of the outer membrane of Gram-negative bacteria, is widely employed experimentally to induce systemic inflammation. LPS specifically binds to Toll-like receptor 4 (TLR4) on endothelial and immune cells, subsequently activating intracellular signaling cascades such as nuclear factor-kappa B (NF-κB) and mitogen-activated protein kinases (MAPKs). The activation of these pathways enhances the expression of inflammatory mediators including TNF-α, IL-1β, inducible nitric oxide synthase (iNOS), and cyclooxygenase-2 (COX-2) [8,9]. Additionally, LPS-induced oxidative stress arises from ROS generation through NADPH oxidase and mitochondrial dysfunction [8]. Excessive ROS react with NO to form peroxynitrite, further impairing endothelial function by suppressing eNOS activity and reducing the functional availability of NO, ultimately resulting in impaired endothelium-dependent vasorelaxation and heightened susceptibility to vascular injury [10,11,12]. Thus, LPS administration serves as an experimental model to reproduce inflammation-associated endothelial dysfunction, which is a key pathological feature underlying diverse cardiovascular diseases.

**Figure 1:**
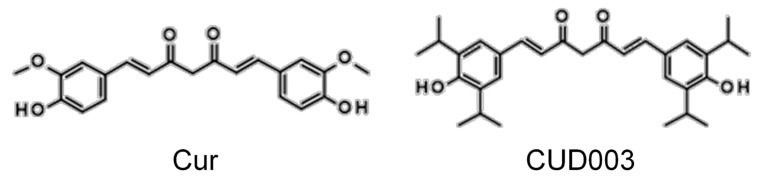
Chemical structures of Cur and CUD003.

Curcumin (Cur; Figure 1), the principal polyphenolic compound found in turmeric (*Curcuma longa*), has been extensively investigated for its potent antioxidant and anti-inflammatory properties, demonstrating beneficial effects in various disease models [13,14,15]. Cur reduces the inflammatory mediators such as TNF-α, IL-6, and COX-2 by suppressing inflammation-related signaling pathways, including NF-κB and MAPKs [15,16,17]. In addition, Cur exerts protective effects against oxidative stress by activating the nuclear factor erythroid 2-related factor 2 (Nrf2) pathway, thereby enhancing the expression of antioxidant enzymes [15,16]. Previous studies have also reported that Cur attenuates LPS-induced vascular inflammation and oxidative stress in animal models [18] and cultured vascular smooth muscle cells [19], and it improves the LPS-induced impairment of vascular responses [20]. Notably, similar bioactivities have also been reported for structurally related diarylheptanoids, highlighting their broad anti-inflammatory and antioxidant potential [21,22]. To further explore structure–activity relationships and to potentially enhance the beneficial effects of Cur, we synthesized four derivatives (CUD001–CUD004) using a method adapted from a previously reported procedure [23]. These derivatives underwent initial in vitro screening in PC12 cells to evaluate baseline cytotoxicity and cytoprotective activity against hydrogen peroxide (H_2_O_2_)- and LPS-induced cell death. Among them, CUD003 (Figure 1) demonstrated the most favorable profile, characterized by low intrinsic cytotoxicity and the highest cytoprotective efficacy under both oxidative and inflammatory conditions (see Supplementary Materials). Based on these results, CUD003 was selected as the lead candidate for in vivo evaluation.

In this study, we, thus, investigated the protective effects of CUD003 against LPS-induced endothelial dysfunction in the thoracic aorta of mice. Vascular function was first assessed by measuring vasoconstrictor responses to phenylephrine (PE), which activates α_1_-adrenergic receptors on vascular smooth muscle to induce contraction. We then examined endothelium-dependent relaxation induced by acetylcholine (ACh), which stimulates muscarinic receptors on endothelial cells to release NO, and endothelium-independent relaxation induced by sodium nitroprusside (SNP), an NO donor that directly relaxes vascular smooth muscle. Together, these assays provide a comprehensive assessment of both endothelial and vascular smooth muscle function, enabling us to distinguish endothelial dysfunction from alterations in smooth muscle responsiveness. The expression of eNOS was evaluated by immunohistochemistry to determine the status of endothelial NO production. Inflammatory responses were examined via the immunohistochemical analysis of TNF-α and COX-2. Oxidative stress was assessed by detecting superoxide generation using dihydroethidium (DHE) staining and by measuring malondialdehyde (MDA) levels as an index of lipid peroxidation. We hypothesized that CUD003 would restore endothelium-dependent relaxation by reducing inflammation and oxidative stress more effectively than Cur.

## 2. Results

### 2.1. Survival Rates and Body Weight Changes

As shown in Table 1, LPS administration (10 mg/kg, i.p.) had little effect on 24 h survival rates. All groups, including those treated with LPS alone or in combination with either CUD003 or Cur, maintained a 100% survival rate, except for the Cur (3 mg/kg) + LPS group, which showed a slightly reduced survival rate (93.3%; 14/15), although the cause of death remains unclear. LPS administration significantly reduced body weight compared to the control group (*p* < 0.01). CUD003 treatment (3 or 10 mg/kg) tended to mitigate this LPS-induced weight loss, but these effects did not achieve statistical significance compared to the LPS group.

### 2.2. Restoration of LPS-Impaired Endothelium-Dependent Vasorelaxation by CUD003

**Figure 2:**
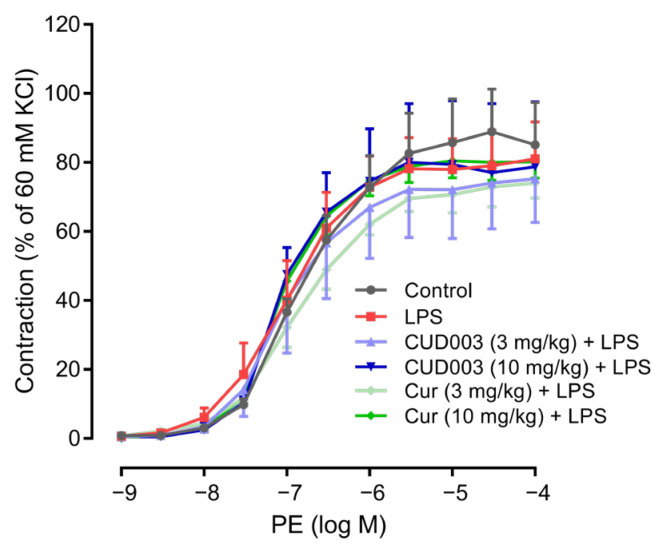
Effects of CUD003 pretreatment on vasoconstriction in aortic rings isolated from LPS-treated mice. Concentration–response curves to PE are expressed as a percentage of the maximum contraction induced by 60 mM KCl. Data are expressed as means ± S.E.M. (*n* = 4–6 per group).

To evaluate the effects of CUD003 on vascular reactivity in LPS-injected mice, we first assessed contractile responses to PE in isolated aortic rings. PE-induced vasoconstriction was expressed as a percentage of the maximum contraction induced by high-potassium (60 mM KCl) solution. As shown in Table 2, the absolute values of 60 mM KCl-induced contraction were comparable among the groups. No significant differences were observed among the groups, indicating that LPS administration did not affect vascular smooth muscle contractility mediated via α_1_-adrenergic receptors (Figure 2).

**Figure 3:**
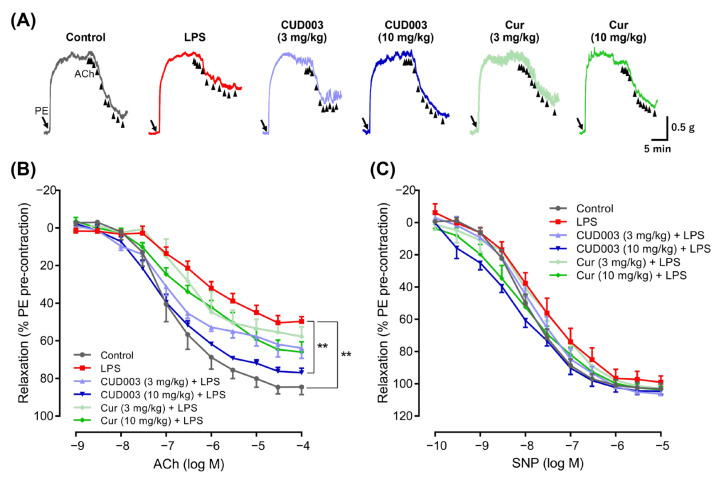
Effects of CUD003 pretreatment on endothelium-dependent relaxation in response to ACh and endothelium-independent relaxation in response to SNP in aortic rings isolated from LPS-treated mice. (**A**) Representative traces showing PE-induced precontraction (arrow) followed by cumulative additions of ACh (black triangles; 10^−9^ to 10^−4^ M), which elicited a stepwise relaxation in each group. (**B**) Concentration–response curves to ACh. (**C**) Concentration–response curves to SNP. Data are expressed as means ± S.E.M. (*n* = 4–6 per group). ** *p* < 0.01.

Subsequently, endothelium-dependent vasorelaxation was examined through the cumulative addition of ACh following PE-induced precontraction. PE-induced precontraction was comparable among the groups (Table 2). Representative traces (Figure 3A) illustrate that, after a stable PE-induced precontraction, the cumulative addition of ACh elicited a stepwise relaxation, as quantified in Figure 3B. These traces demonstrate that LPS administration attenuated ACh-induced relaxation and that this impairment was effectively prevented by pretreatment with CUD003 (10 mg/kg), whereas Cur (10 mg/kg) produced only a modest improvement. LPS administration markedly impaired ACh-induced relaxation compared to the control group (Emax: 83.8 ± 3.9% vs. 51.3 ± 2.8%, *p* < 0.01; Figure 3A,B). Pretreatment with 3 mg/kg CUD003 tended to ameliorate the impaired relaxation (Emax: 65.3 ± 7.8%), although the effect did not reach statistical significance. Notably, pretreatment with CUD003 at 10 mg/kg significantly prevented the impaired relaxation compared to the LPS group (Emax: 76.4 ± 4.2%, *p* < 0.05 vs. LPS), whereas Cur at the same dose showed a non-significant improvement (Emax: 67.7 ± 4.6%). Additionally, the absence of significant differences in endothelium-independent relaxation induced by SNP among the groups (Figure 3C) indicates that the attenuated ACh-induced vasorelaxation was attributable to endothelial dysfunction rather than to alterations in vascular smooth muscle function.

These effects were also reflected in the pD_2_ values (Table 2). LPS markedly reduced the pD_2_ for ACh-induced relaxation compared to the control (7.60 ± 0.17 vs. 6.28 ± 0.20, *p* < 0.01), and this reduction was partially reversed by CUD003 treatment (7.05 ± 0.20, *p* < 0.05 vs. LPS). In contrast, no significant differences were observed in the pD_2_ values for SNP-induced relaxation or PE-induced contraction among the experimental groups.

In summary, LPS impaired endothelium-dependent relaxation without affecting vasoconstrictor responses or endothelium-independent relaxation induced by SNP, indicating that the vascular dysfunction was primarily attributable to endothelial impairment. Pretreatment with CUD003 prevented this impairment, thereby demonstrating its protective effect on endothelial function.

### 2.3. Restoration of LPS-Induced Loss of eNOS Expression in the Thoracic Aorta by CUD003

**Figure 4:**
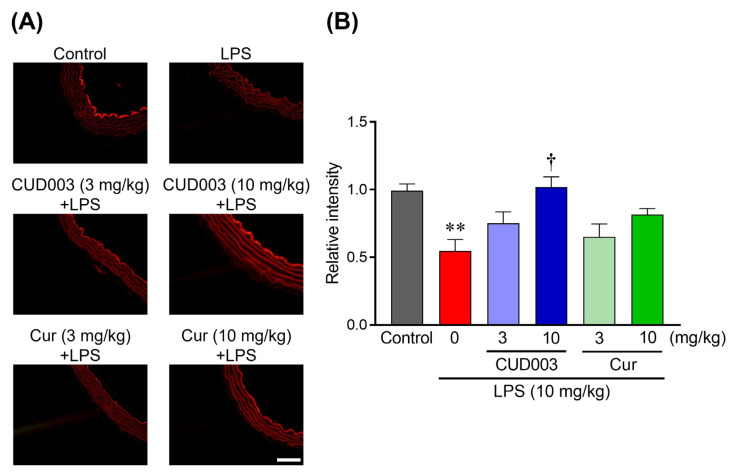
Effects of CUD003 pretreatment on eNOS expression in the thoracic aorta isolated from LPS-treated mice. (**A**) Representative images of immunofluorescence staining for eNOS in aortic sections from each group; scale bar = 50 µm. (**B**) The values of fluorescence intensity of each group are represented as means ± S.E.M. relative to those of the control group (*n* = 5–6 per group). ** *p* < 0.01 vs. control group; ^†^ *p* < 0.05 vs. LPS group.

To evaluate eNOS expression in the thoracic aorta, immunofluorescence staining was performed. In the control group, eNOS protein was distinctly observed along the vascular endothelium, whereas LPS injection significantly reduced its expression level (Figure 4A,B; *p* < 0.01 vs. control). Pretreatment with CUD003 at 10 mg/kg significantly preserved eNOS expression (*p* < 0.05 vs. LPS), while Cur at the same dose showed a trend toward preservation without reaching statistical significance. The lower dose (3 mg/kg) of either CUD003 or Cur did not substantially affect eNOS expression.

### 2.4. Inhibition of LPS-Induced Inflammatory Responses in the Thoracic Aorta by CUD003

**Figure 5:**
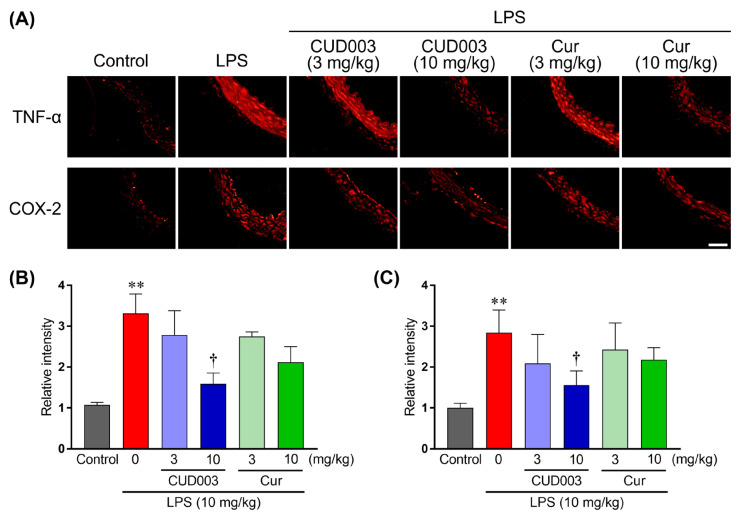
Effects of CUD003 pretreatment on TNF-α and COX-2 expression in the thoracic aorta isolated from LPS-treated mice. (**A**) Representative images of TNF-α (upper) and COX-2 (lower) immunostaining in the thoracic aorta from each group; scale bar = 50 µm. The values of fluorescence intensity of TNF-α (**B**) and of COX-2 (**C**) of each group are represented as means ± S.E.M. relative to those of the control group (*n* = 5–6 per group). ** *p* < 0.01 vs. control group; ^†^ *p* < 0.05 vs. LPS group.

LPS activates TLR4–NF-κB/MAPK signaling and upregulates pro-inflammatory cytokines and the inducible enzyme COX-2, thereby promoting endothelial dysfunction. To investigate the effects of CUD003 on LPS-induced vascular inflammation, immunofluorescence staining for TNF-α and COX-2 was performed in the thoracic aorta (Figure 5). In comparison to the control group, LPS administration markedly increased the expression levels of both TNF-α and COX-2 throughout the vascular wall (Figure 5B,C; *p* < 0.01 vs. control), indicating a pronounced inflammatory response involving cytokine and enzyme-mediated pathways. Pretreatment with CUD003 at 10 mg/kg significantly reduced the LPS-induced upregulation of these inflammatory markers (Figure 5B,C; *p* < 0.05 vs. LPS), indicating a potent anti-inflammatory effect. Cur at the same dose showed a trend toward reduction, although this effect did not reach statistical significance. The lower dose (3 mg/kg) of either CUD003 or Cur had no substantial effect on the expression of these markers.

### 2.5. Inhibition of LPS-Induced ROS Generation and Lipid Peroxidation in the Aorta by CUD003

**Figure 6:**
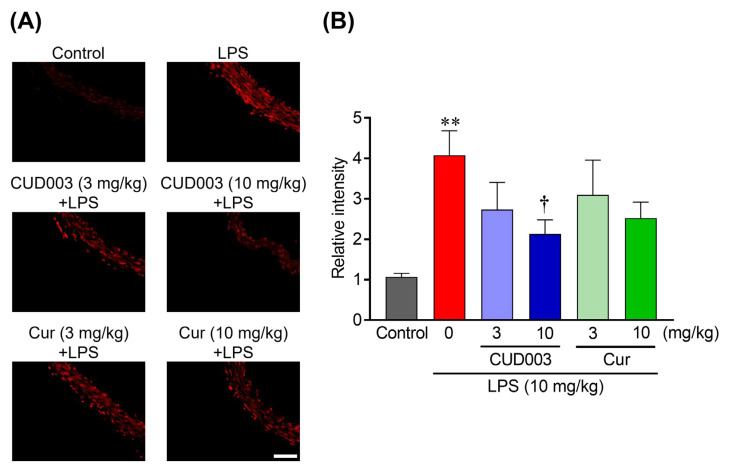
Effects of CUD003 pretreatment on oxidative stress in the thoracic aorta of LPS-treated mice. (**A**) Representative images of dihydroethidium (DHE) staining showing superoxide production in the aorta; scale bar = 50 µm. (**B**) The values of fluorescence intensity of each group are represented as means ± S.E.M. relative to those of the control group (*n* = 5–6 per group). ** *p* < 0.01 vs. control group; ^†^ *p* < 0.05 vs. LPS group.

**Figure 7:**
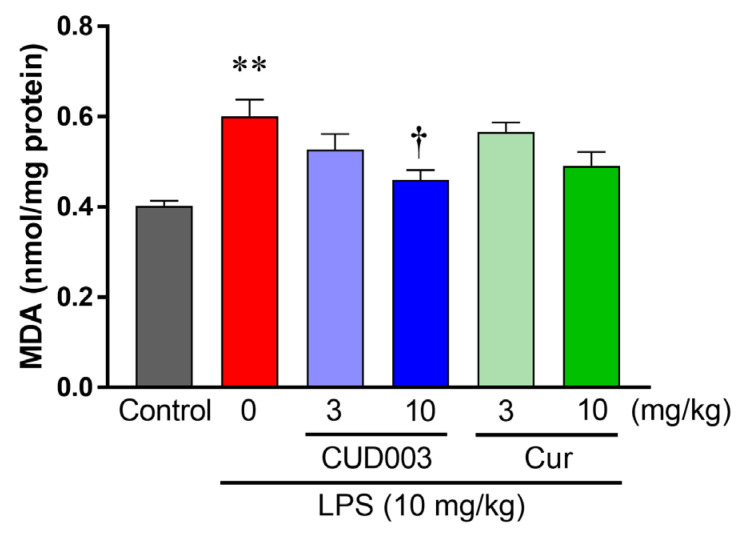
Effects of CUD003 pretreatment on malondialdehyde (MDA) levels in the thoracic aorta of LPS-treated mice. MDA was quantified by the thiobarbituric acid-reactive substances (TBARS) method (MDA–TBA adduct). Data are expressed as means ± SEM (*n* = 5–6 per group). ** *p* < 0.01 vs. control group; ^†^ *p* < 0.05 vs. LPS group.

Inflammatory activation by LPS is accompanied by increased vascular oxidative stress, including ROS generation and lipid peroxidation, which can exacerbate endothelial dysfunction. To evaluate oxidative stress in the thoracic aorta following LPS administration, we assessed intracellular superoxide (O_2_^−^) generation using DHE staining (Figure 6A). In comparison to the control group, DHE fluorescence intensity was markedly increased throughout the vascular wall in the LPS group (Figure 6B; *p* < 0.01 vs. control), indicating enhanced O_2_^−^ production. Pretreatment with CUD003 at 10 mg/kg markedly attenuated DHE fluorescence compared to the LPS group, indicating suppression of LPS-induced oxidative stress. Quantitative analysis revealed that O_2_^−^ production was significantly reduced in the CUD003 (10 mg/kg) group (Figure 6B; *p* < 0.05 vs. LPS), whereas Cur at the same dose exhibited a modest and not statistically significant reduction. To further evaluate oxidative damage, we measured MDA levels in the thoracic aorta as an index of lipid peroxidation. MDA levels, measured by the thiobarbituric acid-reactive substances (TBARS) assay (MDA–TBA adduct), were significantly elevated in the LPS group compared to the control group (Figure 7; *p* < 0.01), indicating enhanced lipid peroxidation. Notably, pretreatment with CUD003 (10 mg/kg) significantly suppressed the LPS-induced elevation in MDA levels (Figure 7; *p* < 0.05 vs. LPS), further corroborating its antioxidant capacity.

### 2.6. Antioxidant Properties of CUD003

We next evaluated antioxidant activity using two cell-free assays. The DPPH assay quantifies single-electron/hydrogen-atom-transfer-based radical scavenging; a decrease in DPPH absorbance reflects scavenging capacity. The TBARS assay monitors the inhibition of lipid peroxidation by measuring the formation of the MDA–TBA adduct. For both metrics, lower EC_50_ (DPPH) and lower IC_50_ (TBARS) indicate greater antioxidant potency. In the DPPH assay, Cur exhibited significantly greater radical-scavenging activity than CUD003, as indicated by a lower EC_50_ value (Table 3; *p* < 0.01). In contrast, both compounds exhibited comparable inhibitory effects in the TBARS assay, showing no significant difference in IC_50_ values (Table 3). Overall, CUD003 exhibited antioxidant activity comparable to, or slightly weaker than, Cur across these in vitro assays.

### 2.7. In Silico ADMET Predictions

Table 4 summarizes the in silico predictions of pharmacokinetic properties and ADMET profiles for CUD003 and Cur, obtained from SwissADME (University of Lausanne and SIB Swiss Institute of Bioinformatics, Lausanne, Switzerland) and pkCSM (University of Queensland, Brisbane, Australia). In silico ADMET predictions indicated that CUD003 exhibits increased lipophilicity and decreased water solubility in comparison to Cur. Despite its lower water solubility, CUD003 was predicted to have higher Caco-2 permeability and intestinal absorption, both of which are potentially attributable to its reduced topological polar surface area. Both compounds were predicted to have low blood–brain barrier permeability, suggesting minimal central nervous system penetration. The volume of distribution for CUD003 was slightly lower than that of Cur, suggesting that CUD003 may predominantly remain in systemic circulation. Predicted interactions with metabolic enzymes and toxicity profiles were comparable between the two compounds.

## 3. Discussion

In the present study, we demonstrated that pretreatment with the novel Cur derivative CUD003 effectively attenuated LPS-induced vascular endothelial dysfunction in the thoracic aorta. Specifically, the endothelium-dependent relaxation response to ACh was significantly impaired in the LPS group, whereas CUD003 administration markedly improved this dysfunction. Furthermore, LPS administration led to a reduction in eNOS expression, the upregulation of inflammatory markers including TNF-α and COX-2, and elevated oxidative stress, as evidenced by increased DHE fluorescence and MDA levels in the aorta. Pretreatment with CUD003, rather than Cur, significantly suppressed all these LPS-induced pathological changes. These protective effects were especially evident at a dose of 10 mg/kg and appeared more potent than those of Cur at the same dose. Collectively, these findings suggest that CUD003 confers vascular protective effects against LPS-induced endothelial dysfunction, possibly by inhibiting inflammation and oxidative stress, thereby preserving vascular endothelial function.

In the present study, only the endothelium-dependent relaxation response to ACh was impaired by LPS treatment (Figure 3A,B), whereas SNP-induced endothelium-independent relaxation and PE-induced vasoconstriction responses remained unaffected (Figure 2 and Figure 3C). These results suggested that the vascular impairment observed in LPS-treated mice was attributable to endothelial dysfunction, rather than to alteration in vascular smooth muscle function. This observation is consistent with previous reports demonstrating that LPS selectively impairs endothelium-dependent vasodilation without affecting smooth muscle contractility [24,25,26]. Endothelium-dependent relaxation is primarily mediated by eNOS-derived NO, and the downregulation of eNOS expression is known to significantly impair this vasodilatory response [27]. Consistent with these findings, we observed a marked decrease in eNOS expression in thoracic aortas from LPS-treated mice (Figure 4), coinciding with impaired endothelium-dependent relaxation. Although our findings are consistent with prior reports in the rodent thoracic aorta, endothelial signaling varies across vascular beds and species; therefore, extrapolation to resistance arteries or human vessels should be undertaken with caution.

Inflammation and oxidative stress play a central role in the development and progression of vascular endothelial dysfunction, acting in a mutually exacerbating manner. LPS activates TLR4, which, in turn, stimulates the NF-κB and MAPK signaling pathways, leading to increased expression of inflammatory mediators such as TNF-α and COX-2 [9,28]. This inflammatory cascade results in endothelial cell activation and the upregulation of adhesion molecules, including intercellular adhesion molecule 1 (ICAM-1) and vascular cell adhesion molecule 1 (VCAM-1), which promote leukocyte adhesion and migration across the endothelium, exacerbating vascular inflammation [1,7,29]. Such inflammatory activation disrupts the vascular endothelial barrier by downregulating tight junction proteins such as claudin-5 and occludin, leading to increased vascular permeability, edema, and inflammation [30]. Disruption of the endothelial barrier also contributes to endothelium-dependent vascular dysregulation. Furthermore, LPS-induced activation of NADPH oxidase results in excessive production of ROS, including O_2_^−^, which reacts with NO to form peroxynitrite, thereby reducing NO bioavailability. Peroxynitrite, a highly reactive and cytotoxic oxidant, contributes to endothelial injury by inhibiting both the expression and activity of eNOS, ultimately impairing vasorelaxation [11]. ROS also promote lipid peroxidation and structural damage to endothelial cells, further accelerating endothelial dysfunction. In this regard, our findings demonstrated that pretreatment with CUD003 significantly attenuated the LPS-induced upregulation of TNF-α and COX-2 expression (Figure 5). Moreover, CUD003 markedly suppressed O_2_^−^ accumulation, as evidenced by reduced DHE fluorescence, and it lowered MDA levels in the thoracic aorta (Figure 6 and Figure 7), indicating a pronounced antioxidant effect in vivo. Importantly, CUD003 significantly prevented the LPS-induced reduction in eNOS expression (Figure 4), suggesting that the observed improvement in endothelium-dependent relaxation is at least partly due to preserved NO production capacity. It should be noted that, although our study evaluated O_2_^−^ generation, lipid peroxidation, and eNOS expression, direct measurement of peroxynitrite was not conducted. Since peroxynitrite is a critical product of the reaction between O_2_^−^ and NO, further studies incorporating peroxynitrite detection, for example, via the immunohistochemical analysis of nitrotyrosine, will be essential to fully elucidate the mechanisms of oxidative vascular injury.

In the present study, Cur at the tested doses showed a dose-dependent trend toward improvement; however, these effects did not reach statistical significance. It is plausible that higher doses of Cur might yield significant protective effects. Notably, CUD003 exhibited significant protective effects at equivalent doses, suggesting greater potency. Collectively, our findings indicated that the structural modification of Cur into CUD003 successfully enhanced its pharmacological efficacy, enabling beneficial outcomes at lower doses. The doses of CUD003 and Cur used in the present study (3 and 10 mg/kg, p.o.) were selected based on our preliminary experiments, which demonstrated preservation of vascular function, as well as on previous reports showing the vascular protective effects of Cur in LPS-induced inflammatory models within similar dose ranges [18,20,31]. These doses were considered appropriate for evaluating comparative efficacy under safe and pharmacologically relevant conditions. The present results, however, suggest that higher doses of Cur may be required to achieve statistically significant protection, whereas CUD003 elicited such effects at the same doses.

Notably, in silico ADMET prediction analyses revealed that CUD003 possesses improved pharmacokinetic properties compared to Cur. Specifically, CUD003 exhibited higher lipophilicity (LogP), increased Caco-2 cell permeability, and enhanced predicted intestinal absorption (Table 4). These advantages are likely attributable to its lower topological polar surface area (TPSA), which facilitates passive transcellular diffusion across lipid membranes, a critical factor for oral bioavailability and cellular uptake [32]. In addition, the predicted volume of distribution for CUD003 was lower than that of Cur, suggesting a tendency for CUD003 to remain predominantly in the plasma rather than being widely distributed to peripheral tissues. This pharmacokinetic profile may contribute to higher local concentrations near vascular endothelial cells, thereby enhancing its efficacy in preventing vascular dysfunction. However, it is noteworthy that the predicted permeability of CUD003 remains below the generally accepted threshold for high intestinal absorption (typically >1 × 10^−6^ cm/s) [33]. Therefore, further structural modifications or advanced formulations might be required to fully optimize its pharmacokinetic profile. While improved pharmacokinetic properties such as enhanced absorption and membrane permeability might partly account for the superior in vivo efficacy of CUD003, they are unlikely to be the sole contributors. Curcumin has been shown to exert potent anti-inflammatory and antioxidant effects, primarily through the suppression of NF-κB signaling and activation of the Nrf2–ARE (antioxidant response element) pathway [34,35]. Although CUD003 exhibited comparable antioxidant potency to Cur in vitro (Table 3), its enhanced in vivo effects may also reflect structural modifications that could confer higher affinity or selectivity toward these molecular targets. However, direct pathway engagement was not examined here. Clarifying whether CUD003 modulates NF-κB or Nrf2 will require pathway-specific assays.

Although in silico predictions indicated comparable metabolic and toxicological profiles between CUD003 and Cur, a potential concern regarding hepatotoxicity was noted. In the present single-administration study, no overt signs of hepatotoxicity (e.g., weight loss, morbidity, or mortality) were observed in any treatment group; however, no direct assessments of liver injury, such as serum transaminase levels or histopathology, were performed. Available toxicological data indicate that structurally related Cur derivatives generally exhibit favorable safety profiles. For example, Activated Curcumin C3 Complex (AC3^®^; Sabinsa Corporation, East Windsor, NJ, USA) showed no adverse effects in rats at doses up to 500 mg/kg/day during 90-day repeated-dose studies [36], and the Cur–galactomannoside complex (CGM; Akay Natural Ingredients Pvt. Ltd., Kochi (Cochin), Kerala, India) was well tolerated in healthy volunteers at doses up to 1000 mg/day for 90 days [37]. Although Cur itself has an established safety profile [38], several cases of hepatotoxicity have been associated with high-bioavailability and high-dose formulations [39]. Given these considerations, future studies should incorporate comprehensive toxicological assessments, with particular emphasis on evaluating chronic hepatic safety, before progressing toward clinical application.

Taken together, our results demonstrate that CUD003 ameliorates LPS-induced endothelial dysfunction through the inhibition of inflammation and oxidative stress, which, in turn, leads to the restoration of eNOS expression. These findings highlight the preventive potential of CUD003 in protecting vascular integrity under inflammatory conditions. The predicted improvements in pharmacokinetic properties, particularly enhanced absorption and plasma retention, may partially account for the superior in vivo efficacy of CUD003. However, an important limitation of the present study is that we only evaluated its effects under preventive conditions, and it remains unclear whether CUD003 can also improve endothelial dysfunction once it has already developed. To support its potential clinical application, future studies should investigate post-treatment paradigms to validate its therapeutic efficacy, but also conduct comprehensive pharmacokinetic, pharmacodynamic, and safety assessments as well as pathway-specific assays to evaluate NF-κB and Nrf2 engagement.

## 4. Materials and Methods

### 4.1. Reagents

CUD003 was synthesized in collaboration with the Laboratory of Pharmaceutical Chemistry at Josai University. Its chemical structure was confirmed by ^1^H and ^13^C NMR spectroscopy and mass spectrometry. The compound’s purity was determined by high-performance liquid chromatography and was found to exceed 95%. Acetylcholine chloride, dihydroethidium, Kolliphor^®^ HS15 (HS15), lipopolysaccharides from Escherichia coli (LPS; 0111: B4), and phenylephrine hydrochloride were purchased from Sigma-Aldrich (St Louis, MO, USA). Curcumin (Cur), dimethyl sulfoxide (DMSO), and sodium nitroprusside dehydrate were purchased from FUJIFILM Wako Pure Chemical (Osaka, Japan). All reagents were dissolved in saline and concentrations are expressed as the final molar concentration in the ex vivo organ bath experiments. Stock solutions of Cur and CUD003 were prepared in DMSO and diluted on the day of the experiment with 2 volumes of HS15 and 17 volumes of distilled water (resulting in a final composition of 1/20 DMSO, 2/20 HS15, and 17/20 water).

### 4.2. Animals

A total of 95 adult male ICR mice (6 weeks old, 28–30 g) were purchased from Japan SLC, Inc. (Hamamatsu, Japan). The mice were housed in groups of between three and four per cage under controlled environments (temperature: 23 ± 0.5 °C; humidity: 55 ± 10%) with a 12 h light/dark cycle (lights on at 7:00 am). Animals had free access to standard rodent chow (CE-2, CLEA Japan, Inc., Tokyo, Japan) and water ad libitum. All mice were acclimated to the environment for 1 week.

### 4.3. Treatment

Mice were randomly divided into six groups: (1) control group (control): mice were orally administered vehicle 30 min prior to an intraperitoneal injection of saline; (2) LPS group (LPS): mice received oral vehicle 30 min before a single intraperitoneal injection of LPS (10 mg/kg); (3) 3 mg/kg CUD003 + LPS group (CUD003 (3)): mice were pretreated with CUD003 (3 mg/kg, p.o.) 30 min prior to LPS administration; (4) 10 mg/kg CUD003 + LPS group (CUD003 (10)): mice were pretreated with CUD003 (10 mg/kg, p.o.) 30 min prior to LPS administration; (5) 3 mg/kg Cur + LPS group (Cur (3)): mice were pretreated with Cur (3 mg/kg, p.o.) 30 min before LPS administration; (6) 10 mg/kg Cur + LPS group (Cur (10)): mice were pretreated with Cur (10 mg/kg, p.o.) 30 min before LPS administration. The dose of LPS was selected based on previous studies [27]. The doses of CUD003 and Cur (3 and 10 mg/kg) were selected based on our preliminary experiments and previous studies demonstrating vascular protective effects of Cur in similar LPS-induced inflammatory models [18,20,31].

### 4.4. Measurement of Vascular Reactivity

Twenty-four hours after LPS injection, mice were deeply anesthetized with isoflurane and decapitated. The thoracic aorta was quickly excised, cleaned of the connective tissue, and cut into 5 mm long rings under a stereomicroscope. We selected the mouse thoracic aorta as a representative conduit artery because it provides stable ring preparations that allow for reproducible contraction and relaxation responses, and it provides sufficient tissue for histological/biochemical assays. Aortic ring fragments were mounted in the organ bath filled with 10 mL of Krebs–Henseleit solution (KHS; 118 mM NaCl, 4.7 mM KCl, 2.5 mM CaCl_2_, 1.2 mM MgSO_4_, 1.2 mM NaH_2_PO_4_, 25 mM NaHCO_3_, 11 mM glucose; pH 7.4) bubbled with 95% O_2_-5% CO_2_ at 37 °C. The aortic rings were attached to a tissue holder and isometric transducer (MLT0202, Panlab, Barcelona, Spain). Isometric tension was amplified (ISO 150-A, Panlab, Barcelona, Spain) and was recorded using the PowerLab data acquisition system (ADInstruments, Castle Hill, Australia) and analyzed with LabChart 8 software (ADInstruments, Castle Hill, Australia). Each aortic ring was equilibrated for 60 min at a resting tension of 1 g before experiments. To evaluate vasoconstriction response, contraction was evaluated by cumulative increases in the concentration of phenylephrine (PE, 1 × 10^−9^ to 1 × 10^−4^ M), and the contractile force was expressed as a percentage of the contraction induced by 60 mM KCl. To assess vasorelaxation responses, the rings were precontracted with PE (1 × 10^−6^ M). Once a stable contraction plateau was reached, cumulative concentrations of acetylcholine (ACh, 1 × 10^−9^ to 1 × 10^−4^ M) or sodium nitroprusside (SNP, 1 × 10^−10^ to 1 × 10^−5^ M) were added to evaluate endothelium-dependent and -independent relaxation, respectively [27].

### 4.5. Immunohistochemistry

Mice were euthanized 24 h after LPS injection, and their thoracic aortas were rapidly excised and frozen in an organic solvent mixture (pentane: hexane, 1:2) at −100 °C using a sample freezer for cryostat (UT2000F, Leica, Bensheim, Germany). Coronal sections (10 µm thick) were prepared using a cryostat (CM3050S, Leica, Bensheim, Germany). The sections were mounted onto glass slides, fixed with methanol for 1 min, and rinsed with 0.01 M phosphate-buffered saline (PBS). After fixation, the sections were preincubated with 5% normal goat serum (Jackson Immuno Research, West Grove, PA, USA) for 1 h at room temperature to block nonspecific binding. Subsequently, the sections were incubated overnight at 4 °C with one of the following primary antibodies: anti-TNF-α antibody (1:100; ALX-210-335, Enzo Life Sciences, Farmingdale, NY, USA), anti-COX-2 antibody (1:100; ab-15191, Abcam, Burlingame, CA, USA), or anti-eNOS mouse monoclonal antibody (1:100; #32027, Cell Signaling Technology, Danvers, MA, USA). After washing three times with PBS, the sections were incubated with secondary antibodies (Cy3; 1:100; Chemicon International, Billerica, MA, USA) for 1 h at room temperature. After washing with PBS, the sections were mounted using VECTASHIELD Mounting Medium (Vector Laboratories, Berkeley, CA, USA). Fluorescent images were captured using an upright microscope (BX53; Olympus, Tokyo, Japan). The fluorescence intensity was quantified using MetaMorph Offline software (version 7.8.1.10.0, Molecular Devices, San Jose, CA, USA) [40]. Histopathological evaluation was performed in a blind manner, without knowledge of the treatment groups.

### 4.6. Analysis of O_2_^−^ Production by Dihydroethidium Staining

Intracellular O_2_^−^ generation in the thoracic aorta induced by LPS challenge was assessed by dihydroethidium (DHE) staining. Cryosections (10 µm thick) were incubated with 10 µM DHE (Sigma-Aldrich, St. Louis, MO, USA) in 10 mM phosphate-buffered saline (PBS, pH 7.4) at 37 °C for 30 min in a dark environment. Excess dye was removed by washing the sections three times with PBS. The sections were then mounted with VECTASHIELD Mounting Medium (Vector Laboratories, Berkeley, CA, USA) and visualized using a fluorescence microscope (BX53; Olympus, Tokyo, Japan). Four areas were randomly selected in each sample, and the fluorescence intensity of oxidized DHE in each field was quantified using MetaMorph Offline software (version 7.8.1.10.0, Molecular Devices, San Jose, CA, USA). The average of the four measurements was calculated and used as the relative fluorescence intensity [40].

### 4.7. Malondialdehyde Assay

Malondialdehyde (MDA) content in thoracic aorta was measured with an MDA assay kit (M496, Dojindo, Kumamoto, Japan) based on the thiobarbituric acid-reactive substances (TBARS) assay to quantify the MDA–TBA adduct, according to the manufacturer’s instructions [41]. Briefly, thoracic aortas were homogenized in PBS containing the antioxidant reagent provided with the kit and centrifuged at 10,000 × *g* for 15 min. The resulting supernatant was mixed with the working solution (prepared according to the manufacturer’s instructions), vortexed, and incubated at 95 °C for 15 min. After cooling on ice, the samples were centrifuged again at 10,000 × *g* for 10 min. The fluorescence intensity of the supernatant was measured using a microplate reader (SpectraMax Pro M5e, Molecular Devices, San Jose, CA, USA) at an excitation wavelength of 540 nm and emission wavelength of 590 nm. Total protein concentration in the tissue homogenates was determined using the Bradford assay with the protein assay dye reagent (Bio-Rad, Berkeley, CA, USA), employing bovine serum albumin (BSA) as the standard.

### 4.8. Free Radical Scavenging and Lipid Peroxidation Inhibitory Activities of CUD003

The free radical scavenging and antioxidative activities of CUD003 and its lead compound, Cur, were evaluated using the 2,2-diphenyl-1-picrylhydrazyl (DPPH) assay and thiobarbituric acid-reactive substances (TBARS) assay, respectively, as previously described [42]. Briefly, test compounds were diluted in ethanol to final concentrations ranging from 5 µM to 1.5 mM. Each diluted sample (100 µL) was added to a 96-well plate followed by 100 µL of DPPH solution (0.2 mg/mL). The mixtures were incubated for 30 min at 25 °C in the dark, and the absorbance was measured at 525 nm using a microplate reader (SpectraMax Pro M5e, Molecular Devices, San Jose, CA, USA). The radical scavenging activity of each compound was expressed as the concentration required to scavenge 50% of DPPH radicals (EC_50_).

The ability of the compounds to inhibit lipid peroxidation was assessed using the TBARS assay. Briefly, 20 µL of linoleic acid (5 mg/mL) and 20 µL of the test compound (0.1–100 µM) were mixed and incubated at 80 °C for 60 min. The autoxidation reaction was terminated by the addition of 200 µL of butylated hydroxytoluene (20 µM). Subsequently, 200 µL of 8% sodium dodecyl sulfate, 400 µL of distilled water, and 3.2 mL of TBA solution (0.25% in 125 mM phosphate buffer, pH 3.0) were added. The mixture was heated at 95 °C for 15 min, cooled on ice, and extracted with 4 mL of ethyl acetate. After vigorous mixing and centrifugation at 2000 rpm for 10 min, the fluorescence intensity of the supernatant was measured using a fluorometer (Wallac ARVO 1420, PerkinElmer, Waltham, MA, USA). Antioxidant activity was expressed as the concentration required to inhibit 50% of lipid peroxidation (IC_50_).

### 4.9. In Silico Absorption, Distribution, Metabolism, Excretion, and Toxicity Prediction

The absorption, distribution, metabolism, excretion, and toxicity (ADMET) profiles of CUD003 were predicted using two publicly available online platforms: SwissADME (http://www.swissadme.ch/ (accessed on 1 July 2025)) and pkCSM (http://biosig.unimelb.edu.au/pkcsm/ (accessed on 1 July 2025)) [43]. The canonical SMILES structures of the compounds were retrieved from PubChem and subsequently used as input for the predictions. SwissADME was utilized to evaluate key physicochemical properties, including lipophilicity, polar surface area (PSA), water solubility, and drug-likeness based on Lipinski’s rules. Additionally, pkCSM was employed to assess several ADMET-related parameters, including Caco-2 cell permeability, human intestinal absorption, blood–brain barrier permeability, volume of distribution, cytochrome P450 (CYP) enzyme inhibition, total clearance, and multiple toxicity endpoints, such as AMES mutagenicity, hepatotoxicity, oral rat acute toxicity (LD_50_), and oral rat chronic toxicity (LOAEL).

### 4.10. Statistical Analysis

All results are expressed as mean ± standard error of mean (S.E.M.). Data analysis of concentration–response curves was performed by two-way analysis of variance (ANOVA) for repeated measurements followed by Tukey’s multiple comparison test. pD_2_ (−log EC_50_) and Emax values were obtained from individual concentration–response curves by nonlinear regression using a four-parameter logistic model (log[agonist] vs. response—variable slope) in GraphPad Prism (version 7.02, GraphPad Software, San Diego, CA, USA). Others were compared by using one-way ANOVA followed by Tukey’s multiple comparisons test. Probability values of *p* < 0.05 were considered statistically significant.

## 5. Conclusions

In conclusion, CUD003, a novel synthetic Cur derivative, ameliorates LPS-induced endothelial dysfunction primarily by inhibiting inflammation and oxidative stress, as demonstrated by reduced TNF-α and COX-2 expression, along with decreased O_2_^−^ production and lipid peroxidation. These effects contributed to the restoration of eNOS expression and improved endothelium-dependent vasorelaxation. CUD003 also exhibited favorable predicted pharmacokinetic properties, such as enhanced intestinal absorption and plasma retention, which might contribute to its superior in vivo efficacy compared to Cur, although these findings require further experimental validation. Collectively, these findings support its potential as a preventive candidate for vascular inflammatory diseases, warranting further pharmacokinetic, pharmacodynamic, and safety studies.

## Figures and Tables

**Table 1:** Survival rates and body weight changes in each group 24 h after LPS administration.

Group	Survival Rates (%)	Body Weight Changes (%)
Control	100 (18/18)	101.2 ± 0.4
LPS	100 (16/16)	90.1 ± 0.4 **
CUD003 (3 mg/kg) + LPS	100 (15/15)	91.2 ± 0.5 **
CUD003 (10 mg/kg) + LPS	100 (15/15)	91.4 ± 0.3 **
Cur (3 mg/kg) + LPS	93.3 (14/15)	90.5 ± 0.5 **
Cur (10 mg/kg) + LPS	100 (16/16)	90.8 ± 0.5 **

** *p* < 0.01 vs. control.

**Table 2:** Emax and pD_2_ values for ACh-induced relaxation, SNP-induced relaxation, and PE-induced contraction in aortic rings isolated from each group.

			LPS			
	Control	LPS	3 mg/kg CUD003	10 mg/kg CUD003	3 mg/kg Cur	10 mg/kg Cur
Contraction (g)						
60 mM KCl	1.3 ± 0.2	1.3 ± 0.2	1.3 ± 0.2	1.4 ± 0.1	1.3 ± 0.2	1.3 ± 0.1
PE	1.0 ± 0.1	1.0 ± 0.1	0.9 ± 0.1	0.9 ± 0.1	0.9 ± 0.1	0.9 ± 0.1
Emax (%)						
PE	82.1 ± 11.7	79.1 ± 9.7	81.2 ± 12.5	78.4 ± 18.3	72.7 ± 5.0	80.2 ± 5.6
ACh	83.8 ± 3.9	51.3 ± 2.8 **	65.3 ± 7.8	76.4 ± 2.4 ^†^	55.7 ± 4.7	67.7 ± 4.6
SNP	106.4 ± 3.4	102.8 ± 6.8	107.1 ± 3.9	108.4 ± 3.1	106.1 ± 4.2	108.4 ± 5.7
pD_2_						
PE	6.80 ± 0.08	6.97 ± 0.17	7.12 ± 0.25	7.11 ± 0.08	6.85 ± 0.14	7.10 ± 0.05
ACh	6.97 ± 0.17	6.28 ± 0.20	6.93 ± 0.17	7.07 ± 0.09	6.52 ± 0.25	6.48 ± 0.11
SNP	7.98 ± 0.11	7.82 ± 0.11	7.99 ± 0.15	8.33 ± 0.15	7.65 ± 0.12	8.14 ± 0.22

Emax: maximum effect; pD2: negative logarithm of the EC_50_; EC_50_: half maximal effective concentration. Data are expressed as means ± S.E.M. (*n* = 4–6 per group). ** *p* < 0.01 vs. control group; ^†^ *p* < 0.05 vs. LPS group.

**Table 3:** Antioxidant activities of each compound via DPPH and TBARS assays.

Compounds	DPPH Assay (EC_50_; µM)	TBARS Assay (IC_50_; µM)
CUD003	81.2 ± 0.7	30.0 ± 3.1
Cur	60.8 ± 1.0 **	29.9 ± 4.3

EC_50_ denotes the concentration producing 50% of the maximal radical scavenging effect; IC_50_ denotes the concentration required to inhibit 50% of MDA–TBA formation under the assay conditions. ** *p* < 0.01 vs. CUD003.

**Table 4:** Pharmacokinetic properties and ADMET prediction of CUD003 using SwissADME and pkCSM.

Parameter	CUD003	Cur
Physicochemical Properties		
Consensus LogP	6.70	3.03
Water solubility (log mol/L)	−4.41	−4.07
Topological polar surface area (Å^2^)	74.60	93.06
Lipinski’s rule	Yes (1 violation)	Yes (0 violation)
Absorption		
Caco-2 permeability (log Papp in 10^−6^ cm/s)	0.382	0.033
Intestinal absorption (% absorbed)	90.6	82.4
P-glycoprotein substrate	Yes	Yes
Distribution		
BBB permeability	−0.113	−0.51
Volume of distribution	−0.65	−0.26
Metabolism		
CYP1A2 inhibitor	No	No
CYP2C9 inhibitor	No	Yes
CYP2C19 inhibitor	Yes	Yes
CYP2D6 inhibitor	No	No
CYP3A4 inhibitor	Yes	Yes
Excretion		
Total clearance (log mL/min/kg)	0.81	0.01
Renal OCT2 substrate	No	No
Toxicity		
AMES toxicity	No	No
Hepatotoxicity	Yes	No
Oral rat acute toxicity (LD_50_; mol/kg)	1.612	1.915
Oral rat chronic toxicity (LOAEL; log mg/kg_bw/day)	1.683	1.586

## Data Availability

The datasets generated and/or analyzed during the current study are included in the article and are available from the corresponding author upon reasonable request.

## Supplementary Materials

The following supporting information can be downloaded at: https://www.mdpi.com/article/10.3390/ijms26188850/s1.

## Abbreviations

The following abbreviations are used in this manuscript:

ACh	Acetylcholine
ADMET	Absorption, distribution, metabolism, excretion, and toxicity
ANOVA	Analysis of variance
ARE	Antioxidant response element
CUD003	Curcumin derivative 003
Cur	Curcumin
COX-2	Cyclooxygenase-2
DHE	Dihydroethidium
DMSO	Dimethyl sulfoxide
DPPH	2,2-diphenyl-1-picrylhydrazyl
EC_50_	Half maximal effective concentration
Emax	Maximum effect
eNOS	Endothelial nitric oxide synthase
HO-1	Heme oxygenase 1
IC_50_	Half maximal inhibitory concentration
ICAM-1	Intercellular adhesion molecule 1
IL	Interleukin
LPS	Lipopolysaccharide
MAPKs	Mitogen-activated protein kinases
MDA	Malondialdehyde
NADPH	Nicotinamide adenine dinucleotide phosphate
Nrf2	Nuclear factor erythroid 2-related factor 2
NF-κB	Nuclear factor kappa B
NQO-1	NAD(P)H quinone dehydrogenase 1
PE	Phenylephrine
O_2_^−^	Superoxide
pD_2_	Negative logarithm of the EC50
ROS	Reactive oxygen species
SNP	Sodium nitroprusside
TBARS	Thiobarbituric acid-reactive substances
TNF	Tumor necrosis factor
TLR	Toll-like receptor
TPSA	Topological polar surface area
VCAM-1	Vascular cell adhesion molecule 1
