# Supplementary material for: CUD003, a Novel Curcumin Derivative, Ameliorates LPS-Induced Impairment of Endothelium-Dependent Relaxation and Vascular Inflammation in Mice
Source: Int J Mol Sci. 2025 Sep 11;26(18):8850. doi: 10.3390/ijms26188850 (PMC12470201; doi:10.3390/ijms26188850)
Supplement: Supplementary file 1 [file ijms-26-08850-s001.zip › ijms-3811210-supplementary materials.pdf]

Supplementary Materials

CUD003, a novel curcumin derivative, ameliorates LPS-induced impairment of endothelium-dependent relaxation and vascular inflammation in mice

Supplementary Result

Restoration of H_2O_2- or LPS-Induced Loss of Cell Viability in PC12 cells by CUD003

To evaluate the cytoprotective effects of CUD003 under oxidative and inflammatory stress conditions, PC12 cells were pretreated with CUD003 or curcumin (1–1000 μM) for 1 hour prior to exposure to either hydrogen peroxide (H_2O_2, 50 μM) for 4 hours or lipopolysaccharide (LPS, 600 μg/mL) for 24 hours. As shown in Figure S1, exposure to H_2O_2 or LPS significantly reduced cell viability compared to untreated control ($p < 0.01$). Pretreatment with CUD003 attenuated H_2O_2- (Figure S1A) and LPS-induced cytotoxicity (Figure S1B) in a concentration-dependent manner ($p < 0.01$ vs. H_2O_2 or LPS alone). Curcumin also exerted protective effects at higher concentrations, but the efficacy was generally lower than that of CUD003 at equivalent doses.

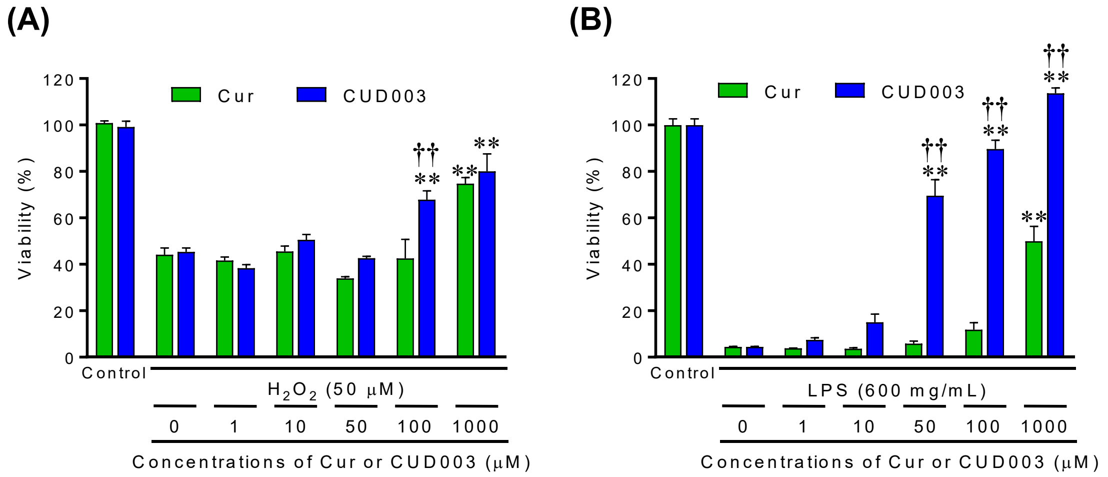

Figure S1. Protective effects of CUD003 against H_2O_2- or LPS-induced cytotoxicity in PC12 cells. PC12 cells were pretreated with CUD003 or curcumin (1–1,000 μM) for 1 h, followed by exposure to 50 μM hydrogen peroxide (H_2O_2) for 4 h (A) or 600 μg/mL lipopolysaccharide (LPS) for 24 h (B). Cell viability was determined using the CellTiter-Glo Luminescent Cell Viability Assay. Data are presented as mean ± SEM. n = 3 per group. ** $p<0.01$ vs. H2O2 alone, †† $p < 0.01$ vs. Cur at equivalent concentration (A); n = 5 per group. ** $p<0.01$ vs. LPS alone, †† $p < 0.01$ vs. Cur at equivalent concentration (B).

Supplementary Materials and Methods

・*Cell Culture*

PC12 (JCRB0268) cells were purchased from the Japanese Collection of Research Bioresources Cell Bank (Osaka, Japan). PC12 cells were cultured in RPMI 1640 medium (GIBCO; Thermo Fisher Scientific, Cat.11875-093, Bohemia, NY, USA) supplemented with 10% donor Horse serum (SAFC Pharma, Carlsbad, CA, USA), 5% fetal bovine serum (Nichirei Biosciences Inc., Tokyo, Japan), and antibiotics (100 unit/mL penicillin, 100 μg/mL streptomycin, 0.25 μg/mL amphotericin (Wako Pure Chemical Industries, Osaka, Japan) in a humidified 5% CO2 atmosphere at 37°C. The cells were seeded in a 10 cm Poly-D-Lysine-coated dish (CORNIG Inc., Corning, ME, USA) and allowed to form an adherent culture as a monolayer.

・*Cell Viability Assay*

PC12 cells were seeded in 2×10^4 cells/well in Poly-D-Lysine-coated 96-well plates (CORNIG Inc., Corning, ME, USA) and cultured for 2 days. To examine the cytoprotective activity of CUD003 against oxidative stress and inflammatory stress, the cells were treated with either CUD003 or Cur ranging from 1 to 1,000 μM for 1 h prior to treatment with 50 μM of H_2O_2 for an additional 4 h or 600 μg/mL of LPS for an additional 24 h. Cell viability was measured using a CellTiter-Glo Luminescent Cell Viability assay kit (Promega, Madison, WI, USA) according to the method previously described[1]

References

1. Asano, T.; Xuan, M.; Iwata, N.; Takayama, J.; Hayashi, K.; Kato, Y.; Aoyama, T.; Sugo, H.; Matsuzaki, H.; Yuan, B.; et al. Involvement of the Restoration of Cerebral Blood Flow and Maintenance of ENOS Expression in the Prophylactic Protective Effect of the Novel Ferulic Acid Derivative FAD012 against Ischemia/Reperfusion Injuries in Rats. *Int. J. Mol. Sci.* **2023**, *24*, doi:10.3390/ijms24119663.

Abbreviations

CUD003: Curcumin derivative 003; Cur: Curcumin; H_2O_2: Hydrogen peroxide; LPS: Lipopolysaccharide.
